# Automated Virtual Reality Cognitive Therapy (gameChange) in Inpatient Psychiatric Wards: Qualitative Study of Staff and Patient Views Using an Implementation Framework

**DOI:** 10.2196/34225

**Published:** 2022-04-12

**Authors:** Poppy Brown, Felicity Waite, Sinead Lambe, Julia Jones, Lucy Jenner, Rowan Diamond, Daniel Freeman

**Affiliations:** 1 Oxford Health NHS Foundation Trust Oxford United Kingdom; 2 Department of Psychiatry University of Oxford Oxford United Kingdom; 3 Nottinghamshire Healthcare NHS Foundation Trust Nottingham United Kingdom

**Keywords:** virtual reality, automated, therapy, inpatient psychiatric care, implementation

## Abstract

**Background:**

Automated virtual reality (VR) therapy could allow a greater number of patients to receive evidence-based psychological therapy. The aim of the gameChange VR therapy is to help patients overcome anxious avoidance of everyday social situations. gameChange has been evaluated with outpatients, but it may also help inpatients prepare for discharge from psychiatric hospital.

**Objective:**

The aim of this study is to explore the views of patients and staff on the provision of VR therapy on psychiatric wards.

**Methods:**

Focus groups or individual interviews were conducted with patients (n=19) and National Health Service staff (n=22) in acute psychiatric wards. Questions were derived from the nonadoption, abandonment, and challenges to the scale-up, spread, and sustainability framework. Expectations of VR therapy were discussed, and participants were then given the opportunity to try out the gameChange VR therapy before they were asked questions that focused on opinions about the therapy and feasibility of adoption.

**Results:**

There was great enthusiasm for the use of gameChange VR therapy on psychiatric wards. It was considered that gameChange could help build confidence, reduce anxiety, and “bridge that gap” between the differences of being in hospital and being discharged to the community. However, it was reflected that the VR therapy may not suit everyone, especially if they are acutely unwell. VR on hospital wards for entertainment and relaxation was also viewed positively. Participants were particularly impressed by the immersive quality of gameChange and the virtual coach. It was considered that a range of staff groups could support VR therapy delivery. The staff thought that implementation would be facilitated by having a lead staff member, having ongoing training accessible, and involving the multidisciplinary team in decision-making for VR therapy use. The most significant barrier to implementation identified by patients and staff was a practical one: access to sufficient, private space to provide the therapy.

**Conclusions:**

Patients and staff were keen for VR to be used on psychiatric wards. In general, patients and staff viewed automated VR therapy as possible to implement within current care provision, with few significant barriers other than constraints of space. Patients and staff thought of many further uses of VR on psychiatric wards. The value of VR therapy on psychiatric wards now requires systematic evaluation.

**International Registered Report Identifier (IRRID):**

RR2-10.2196/20300

## Introduction

### Potential of Virtual Reality Therapy

Virtual reality (VR) has the potential to be used in the treatment of a range of mental health problems [1]. Aside from the evaluation of clinical effects, there also needs to be consideration of successful implementation in services. A setting where VR therapy may be particularly valuable is psychiatric hospital wards. Pressures on staff time can often lead to limited opportunities for patients to receive psychological interventions or other meaningful activities [2,3]. Clinical symptoms may be reduced upon hospital discharge, but patients are often unprepared for returning to the situations that they had found difficult before admission. VR can provide a safe and controlled setting for patients to practice being in everyday situations. We therefore set out to investigate how VR therapy is viewed by patients and staff in psychiatric hospitals [4].

### Objectives

The objectives are 3-fold: first, to obtain initial expectations of patients and staff about using VR headsets and, especially, VR psychological therapy; second, to gain patient and staff views of an automated VR therapy (gameChange). gameChange, which has a user-centered design [5,6], was evaluated in a randomized controlled trial with 346 patients with psychosis [7,8]. Almost all patients were attending outpatient services. The VR therapy led to significant reductions in anxious avoidance and distress, particularly for patients with severe agoraphobic avoidance. In 6 sessions, the aim is to reduce agoraphobic avoidance by presenting graded VR simulations of common everyday situations (eg, getting on a bus and going to a shop) [5,6]. Patients are guided through the program by a virtual coach. The third objective is to consider requirements for implementation. The study design was informed by the nonadoption, abandonment, and challenges to the scale-up, spread, and sustainability (NASSS) implementation framework for health care technologies [9]. Staff and patients were in a position to inform 3 of the framework’s 7 domains with regard to implementation of VR therapy: the condition and disorder that the therapy is designed to address, the intended adopters of VR therapy, and the organization where it would be implemented. This is the first implementation study of automated VR therapy in inpatient settings.

## Methods

The gameChange Lived Experience Advisory Panel (LEAP), facilitated by the McPin Foundation, contributed to the development of the study. Details of this and other aspects of the study methodology are provided in the full study protocol [4].

### Amendment to Protocol

The study was set up before the COVID-19 pandemic. The first focus group was run on March 6, 2020. It had been planned to go on to visit 1-2 inpatient wards at each of 5 National Health Service (NHS) mental health trusts across England, totaling a minimum of 50 participants. However, access to wards and travel across the country became severely restricted. Therefore, we had to reduce the number of sites visited.

### Participants

An acute inpatient ward at Nottinghamshire Healthcare NHS Foundation Trust and 2 acute inpatient wards at Oxford Health NHS Foundation Trust took part in the study. Staff working in either the delivery or management of clinical care on the wards were invited to take part. NHS patients staying on the wards were recruited according to the criteria presented in Textbox 1.

**Inclusion and exclusion criteria.**

**Inclusion criteria**

- Participant is willing and able to give informed consent for participation in the study
- Aged ≥18 years
- Willing to consent to being audio recorded
- Sufficient English language skills to participate in the focus group or interview

**Exclusion criteria**

- High levels of associated risk to self or others through participation in the study; for example, actively suicidal
- Photosensitive epilepsy (use of virtual reality is not recommended for those with photosensitive epilepsy)

### Procedure

Focus groups were the primary choice for data collection, but an individual interview was offered where a participant preferred it or was unavailable at the time of the focus groups. Focus groups and interviews initially asked questions relating to expectations before all participants briefly tried gameChange and then discussed their opinions on the therapy and its suitability for the ward. Participants could choose which VR scenario they wanted to try out and what level, although patients were encouraged to try out easier levels first. Each VR scenario lasts several minutes, with higher levels typically being slightly longer and more participative. Where there was enough time, participants could try more than one level or scenario if they wanted.

### Topic Guide

The semistructured topic guide was informed by the NASSS framework. Separate but similar topic guides were created for staff and patients. Study authors, including qualitative research experts, and the LEAP members developed the first drafts of the patient topic guide, and both guides were piloted beforehand. The topic guide was reviewed after conducting the first focus group. No significant changes were made, although 2 questions were slightly rephrased (eg, “Who would you like to deliver VR therapy to you?” was changed to read “If this were to be available on the ward, who would you like to be doing it with you?”).

### Analysis

Focus groups and individual interviews were audio recorded and transcribed verbatim. Field notes from each focus group and interview were also transcribed. Field notes recorded factors such as group dynamic and nonverbal cues to add context to the transcript of the audio recordings. Transcripts were not returned to participants for comment or correction.

A thematic analysis was performed [10] separately for staff and patient data, although similarities and differences between the analyses were then considered. All data were entered into NVivo (version 12; QSR International) [11] to provide a transparent audit trail. The transcribed data were read and reread to ensure familiarity before developing a preliminary coding framework that was discussed and adapted by the first author (PB) during supervision. A number of transcripts were double coded. An extract of the coding and reflexive log, with examples of adaptations made, can be viewed in Multimedia Appendix 1. Details regarding each code were recorded in memos in NVivo. Themes were derived from the data. Diverse cases and minor themes were considered because breadth was considered as important as frequency.

### LEAP Involvement in the Analysis

A summary of the analysis was sent to the LEAP members for consideration to assess the validity of the findings in an additional group. LEAP members showed considerable support for the findings. In particular, they highlighted the need to have treatments beyond medication, the potential for VR to be a helpful route to engaging patients who may otherwise not engage with ward activities, and the potential to have alternative VR scenarios and presentations of Nic, the virtual therapist. The importance of VR increasing access to psychological therapy, rather than being a substitute for any existing therapeutic activity, was also emphasized. A LEAP member additionally underscored that limited private space to use the VR would likely be a significant challenge facing many wards.

### Reflexivity

All patient focus groups were led by a doctoral student (PB) and cofacilitated by a clinical psychologist (SL, RD, or JJ). All interviews and staff focus groups were either led solely by PB or jointly by PB and a clinical psychologist. Consideration was given to how professional backgrounds may affect data collection and analysis. For example, existing knowledge, expectations, and hopes regarding VR therapy may have affected how the focus groups were conducted. A reflexive log was kept, and to try to minimize these potential biases, the topic guide was closely adhered to because this was created largely from the NASSS implementation framework rather than from personal experience and expectations. Consideration was given to the gender and class of the facilitators and that visible indicators of socioeconomic status could affect participant engagement. Participants were frequently reminded that the aim of the study is to hear and learn from their views and that the facilitators wanted participants to be as honest and open as possible about any concerns or criticisms they may have.

### Ethical Considerations

The study had received ethical approval as part of a substantial amendment to the gameChange trial [7]. The trial received ethical approval from the NHS South Central–Oxford B research ethics committee (19/SC/0075).

## Results

### Overview

In total, 19 patients (n=12, 63% men and n=7, 37% women) and 22 members of ward staff (n=3, 14% men and n=19, 86% women) took part. Participants were from 3 wards across 2 NHS mental health trusts. There were 7 patient interviews and 4 patient focus groups (each with 3 patients) and 3 staff interviews and 4 staff focus groups (each with 2-7 staff members). The numbers of staff and patients recruited from each ward were approximately equal. Participants were predominantly of White ethnicity, with ages ranging from 18 to 60 years for the patient participant group and from 21 to 60 years for the staff participant group. The staff comprised nurses (including clinical leads), health care assistants, a deputy ward manager, a peer supporter, a ward clerk, activity coordinators, occupational therapists, and assistant psychologists. Although analyzed separately to begin with, all themes were shared across staff and patient responses.

### Desire for Treatments Beyond Medication and the Value of Psychological Therapy

Many patients described their dissatisfaction with medication being the primary form of treatment available on their ward and the lack of psychological therapy: “How are we going to get better if we’re just on meds?...I would really benefit from therapy at this point.” [participant 8]; “We just get filled with pills, there’s no talking therapies or anything like that” [participant 3]. This desire for treatment beyond medication led to a sentiment of being “up for trying anything” [participant 1]. Patients typically reported a positive view of psychological therapy and a desire for more to be available: “more one to one therapy” [participant 9]; “I think talking’s the way forward” [participant 11]. However, there were some exceptions, with an individual saying, “I don’t find talking helps” [participant 18] and another individual describing some negative past experiences with a psychologist and suggesting instead that their priority for recovery was seeking safe housing [participant 17]. Notably, many patients were aware of resource limitations contributing to a lack of therapy provision: “The room and the money is obviously not enough” [participant 2]; “They’re under a lot of pressure, you see” [participant 15]. Staff also reported positive views of psychological therapy, seeing it as an important treatment option for patients: “It’s always good to have more therapy” [participant 3]; “the most helpful thing for [patients] to have” [participant 19]. Some staff felt that even if therapy could not lead to large clinical improvements, it would nonetheless help patients to have a purpose while being on the ward and help to reduce boredom. There was acknowledgment from a staff focus group that the psychological perspective differs somewhat from the nursing point of view but both are important.

### VR Therapy Sounds Rational and Helpful

Before trying it for themselves, patients and staff members reported positive expectations of gameChange. In particular, they felt that the use of technology, graded levels of difficulty within the program, and the automation of the therapy could all be beneficial. Several staff reported expecting the VR therapy to be popular among patients and felt that it would likely help a lot of patients: ‘‘It makes perfect sense...it’s definitely something that I think could be really useful...just giving them a bit more confidence” [participant 1]. These views were also shared by patients: “If someone struggles with walking down the street and they can do that in chunks and chunks and chunks and gradually build up, like, that’s going to be great” [participant 11]. However, some patients did express concern. After hearing about the rationale of gameChange, a patient stated, “Sometimes I wonder whether highlighting these areas can make the issue a bigger thing” [participant 14].

### Surpassing Expectations

After trying the gameChange VR therapy for themselves, many staff and patients reported feeling surprised and impressed. In particular, there was considerable discussion by all participants of how surprisingly real the VR therapy felt and how the experience was enjoyable. For instance, a staff member stated, “That was really amazing...it does absorb you into it” [participant 11]. Several participants said that the VR therapy had surpassed their expectations: “It’s better than I thought it would be” [staff member, participant 2]; “I was skeptical before coming in, but I get it now” [patient, participant 3]. Several patients also expressed a desire to try more of it and thought it would be very popular on the wards: “I think there would probably be a big line, a big queue, to use it daily I think, to be honest” [participant 4]. However, a member of the staff reported thinking that the VR therapy actually had a strong “sense of unrealism” and that “nothing much” had surprised them [participant 9].

### VR Therapy Could Help

The expectation that the gameChange VR therapy would be helpful was maintained after participants tried it. Patients felt that the gameChange therapy could help in a number of ways, including building confidence and reducing social anxiety (“I think it would be helpful to people with anxiety...I reckon it would help” [participant 19]), providing new perspectives and an escape from the ward on the ward (“I already feel as though I’ve been out today by just being in that experience, and I actually feel better than when I arrived, so it clearly can help” [participant 1]), and preparing for discharge (“It is going to help you to come out into society, out of the hospital, and back into society” [participant 7]). Staff shared patients’ views that gameChange could help build confidence, reduce anxiety, and “bridge that gap” [participant 2] between hospital and discharge and also felt that the VR would be particularly helpful for patients who may typically engage less in therapeutic activities available on the ward, as well as for those who struggle with communication and those who find it difficult to leave their bedrooms. A staff member who had seen some of the patients on the ward trying out the VR therapy also noted: “Seeing them afterwards they seemed really pleased with themselves and it was that kind of sense of accomplishment that was really nice” [participant 17]. However, staff and patients acknowledged that the therapy would not suit everyone. For example, it was discussed that some patients may be too unwell to use the therapy or feel that it is not relevant to their needs: “When [patients are] really unwell it’s difficult...it would have to be, you know, picked up at the right time in their recovery for it to benefit them” [staff member, participant 8]; “Initially you might not be at the stage to do any talking therapies” [patient, participant 3]. Some patients also said that the therapy would not be of particular help for themselves, even if it would help others: “Social situations as he said, brilliant, but like for self-harming...I can’t see that helping in my situation” [participant 2]; “It’s not beneficial to me but it would be a massive help for others that are struggling” [participant 13].

### Envisioning Implementation

Where the VR could be physically located on the ward, who would support patients to use it, and which patients it might be offered to and when was discussed. Staff and patients thought that the VR needed to be stored away somewhere safe and secure and that a quiet, private room would be needed for using VR for structured therapy interventions such as gameChange. The wards varied as to whether such a space existed already. A staff member suggested that an option to overcome space challenges on the ward would be to have a “dedicated space off the ward to use the [VR therapy]” [participant 4], although this would require patients to be granted leave from the ward, which would not always be possible.

Regarding who would be present to support the patient to use VR therapy, patients and staff stressed the importance of the member of staff being someone the patient could trust and with whom the patient could form a good therapeutic relationship: “someone you feel comfortable around” [patient, participant 5]; “It should be done with somebody that they’ve got that therapeutic relationship with” [staff member, participant 22]. Unsurprisingly, staff spent longer considering which specific job roles may be most suited to using the VR therapy with patients. Suggestions included assistant psychologists, health care assistants, and occupational therapists. Of the 4 staff focus groups, 2 (50%) noted that it might be important to have staff members who do not have to respond to personal infrared transmitter (PIT) alarms for ensuring that sessions are not disrupted: “If someone has got a VR headset on and all of a sudden this massive alarm is going off...the person facilitating has to run out of the room...that could be really disorientating” [participant 5]. Although there was agreement that staff would be “very much willing to be trained in it” [participant 1] and would find it enjoyable to be able to “see the benefits” of the treatment [participant 2], it was considered particularly important to ensure an opt-in system, where staff members could sign up to train in the VR therapy if they wanted to but were not required to if they felt that it was not something they would like to do. It was also suggested that, to begin with, it may be helpful to have staff from outside the ward come and “train the whole ward” [participant 1] or even to deliver the therapy to patients, given that external staff would be “more competent and committed” and could then “get the ward staff involved” [participant 2]. When asked about the possibility of a peer professional, that is, someone with lived experience of a mental health problem who has received training in providing psychological support and confidentiality, being present rather than a member of ward staff, patients saw this as a positive option: “They’d be brilliant” [participant 2]; “They’re then speaking from experience, aren’t they” [participant 4].

Staff members also felt that if VR therapy were to be implemented on the wards, its use by individual patients would need to be discussed within the clinical team and then prescribed in line with the evidence base: “It would have to form part of a care plan...it wouldn’t be something that we just get out and go” [participant 11]. In general, staff mostly felt that the therapy could fit well into existing ward routines: “[Staff] set time aside to sort of have one-to-ones with patients...I think you could incorporate it into that hour” [participant 1]; “I take patients out for, like, community assessments and stuff...so the alternative could be doing this” [participant 12].

### Concerns About Having VR Therapy on Wards

Both staff and patients raised concerns regarding how VR therapy could be implemented on wards, although the specific concerns varied. Patients discussed whether VR would be seen as a burden by staff because of the need for constant supervision (“Staff could see it as an imposition because they’re too busy taking people out on fag breaks” [participant 3]), the headsets getting broken or forgotten about (“It’d get broken” [participant 16]), the therapy becoming a substitute to enable further cuts to funding of existing psychological therapies (“I think the danger of course is that the technology becomes the substitute for government cuts or lack of funding” [participant 1]), as well as needing to ensure that patient data are kept secure and confidential (“I would want to know that my data was secure” [participant 8]). A patient focus group also voiced concern that it could be embarrassing if one were doing something odd in the VR, which tied into desires for using it in a private space with a trusted member of staff. Staff members shared patients’ concern about needing to consider how to look after the equipment and prevent it from getting broken: “I could just see the equipment getting ruined” [participant 21]. Staff also raised concerns around whether the headset might be overwhelming or overstimulating for some patients and difficult for those with less spatial awareness.

### Barriers and Facilitators to Implementation Vision

Staff thought that having ongoing access to training, the involvement of a patient’s multidisciplinary team, and a mechanism for helping patients to continue to use the VR if discharged to the community in the middle of a set of sessions would all be factors that would make it easier to ensure the successful implementation of VR therapy on psychiatric wards. Having a staff member lead the use of VR on the ward, who would, for example, be someone staff members “can report back to with any concerns” [participant 1] and who would be responsible for maintaining the equipment was also raised as a facilitator. In addition, staff and patients stressed the importance of introducing the VR therapy in the right way. Patients primarily spoke about this with regard to how it would be explained to users, for example, providing reassurance regarding its safety, and “explaining it has been developed with people with psychosis” [participant 11] (the gameChange VR therapy was developed with patients using a user-centered design process). Staff primarily considered how it should be explained to staff: “as much information as you could give...why it’s going to benefit, what you hope the outcome will be and basically that it could help create a calmer environment on the ward because that’s all we want” [participant 8].

In contrast, current barriers to the implementation of VR therapy on wards included staff shortages and the resultant reliance on agency staff, as well as the lack of appropriate space for using the VR therapy on some of the wards, with existing private spaces either being too small, too noisy, or too infrequently available. There was contrast among members of staff within and between wards regarding whether limits on staff time would be a problem. Some members of staff felt that the VR therapy would not add time pressure to staff roles because it could fit into existing routines or that any additional time it would require would likely only bring about savings in time in the longer term (“I wouldn’t say the time is a constraint, no, no...if we’re spending more time engaging in therapy with someone that can only be a positive” [participant 1], whereas others felt that pressures on staff time would be a greater challenge, and would, for example, “play a part in how frequently somebody could have a session” [participant 16].

### Improvements and Potential

Several ways of improving the VR therapy were discussed. Patients and staff thought it would be beneficial to be able to vary the computer characters and, in particular, the virtual coach, Nic, to suit the preferences of the user. A patient focus group also suggested that Nic could be presented as a peer professional, for example, “a patient with your own characteristics that’s out in the community” [participant 1], feeling that “if it’s presented as a peer supporter, even though it’s not real I think that would make you feel a little bit more relaxed” [participant 3]. A number of additional scenarios were also suggested, including a football stadium, a theater, a courthouse, and a workplace. Having some simulated ward environments such as the communal area and a ward round meeting were also suggested by several members of staff and patients: “[Patients] can get really anxious about ward rounds...so I don’t know whether or not that could be something in future” [staff member, participant 20]; “a ward meeting where there’s loads of people” [patient, participant 16]. Other improvements suggested were having adaptations for individuals with audio or visual impairments and increasing the level of interactivity in the scenarios. Staff and patients also discussed a range of ideas for further uses of VR headsets. Relaxation and mindfulness exercises were frequently discussed in particular, with other suggestions including helping autistic individuals to practice eye contact; training in STOP anger management techniques; and staff training on what it is like to have certain psychotic experiences, patient assessment and diagnostics, treating posttraumatic stress disorder, and treating obsessional thinking. As a patient stated, “There’s sort of endless possibilities” [participant 1]. Because of the limited resourcing many wards face, it was also suggested that patients could use the VR headsets for gaming when available, which might then also help to reduce boredom on a ward.

## Discussion

### Principal Findings

We report the first qualitative investigation of staff and patient views on the potential of using automated VR cognitive therapy on inpatient psychiatric wards. It was very clear that patients and staff have considerable enthusiasm for trying something new, especially a potentially effective psychological approach, and that participants were impressed by the potential of the automated VR therapy to help patients, while potentially overcoming some of the resourcing challenges that traditional therapies face. Although caveats were expressed, the enthusiasm bodes well for testing and implementing VR therapy on psychiatric wards.

Separate coding frameworks were initially developed, but there was considerable overlap and consensus between patient and staff views. Particularly striking was that nearly all participants felt positively surprised by certain aspects of gameChange, noting that it surpassed their expectations, in particular with regard to how real it felt. In addition, although staff and patients felt that on a patient’s immediate arrival to the ward VR therapy may not be so appropriate, psychological therapy is certainly something that was desired by patients and considered by staff to be important for aiding recovery. Patients staying in hospital may often be thought of as being too unwell to benefit from psychological therapy, but this was not the view of the patients and staff from these wards.

Staff and patient participants both shared the belief that VR therapy could be very helpful, and they were keen to consider practical solutions concerning where and with whom it could be used. There was also variation in the discussion by staff and patients. Within the *envisioning practicalities theme*, staff considered in greater detail which professions might be able to feasibly deliver VR therapy, whereas patients understandably discussed in greater detail who they might feel most comfortable in having to support them. Interestingly, a primary concern of the patients centered on whether staff would be willing and have the time to use the VR therapy with them, whereas many staff members did not raise this as a likely problem.

The topic guide covered three domains of the NASSS framework: the condition or illnesses that the technology is designed to help, the intended adopters of the technology, and the organization where it would be implemented. With regard to the condition, the gameChange automated VR therapy is designed to help anyone who may feel anxious or lack confidence in entering everyday social situations. It is for agoraphobic-type anxious avoidance, which occurs in two-thirds of patients with severe mental health conditions [12]. Patients and staff agreed that this would be a relevant treatment target for many individuals on the ward, but they were of the opinion that factors such as severity of clinical symptoms might complicate successful use. Interestingly, wider applications of VR for patients in psychiatric wards were identified. With regard to the intended adopters, a crucial lesson from this study is the clear enthusiasm and positive feedback displayed by all participants. This is particularly of note, given that studies suggest that acceptance by staff can often be the single most important determinant of whether new technologies succeed at a local level [9,13]. However, it must also be recognized that the staff most likely to volunteer their time to take part in an interview may also be those who judge that they have time available or have the most interest in innovation. Self-selection is likely to bias feedback toward the positive. This potential bias may have been mitigated to a degree by running several focus groups in a regular staff meeting slot. However, it is also the case that most of the staff interviewed were not in senior decision-making roles for ward treatment provision. Regarding the NASSS framework domain of organization, most staff reported that their ward would have the capacity and motivation to take on the kind of change entailed by VR therapy. It was judged that the use of VR therapy could fit into existing ward routines such as one-to-one time that staff have dedicated to spending directly with individual patients, although its use in conjunction with real-life practice in outdoor settings may require careful planning.

A number of potential barriers to implementation were raised. Space to use the equipment may be a barrier in some wards. Staff did think that this barrier could be overcome through adapting current spaces or making use of rooms off the ward. Although staff time was not seen as a barrier when wards are working with usual capacities, times of staff shortages was discussed as a potential problem. This might mean that having staff external to the ward, such as peer professionals dedicated to the delivery of VR therapy in addition to training ward staff, could be the most feasible and popular method of implementing VR therapy. This also fits with recommendations within the NHS Long Term Plan to recruit a workforce of peer support workers in acute settings [14,15].

Our experience is that people need to try VR to understand it fully, and this was the case in this study. For implementation, a VR ward facilitator could ensure that as many staff as possible have the chance to try VR. When introducing the technology to patients, it will be helpful to address explicitly the concerns raised by patients in this study; for example, by providing information on the safety of the equipment and whom it was developed by. Resources such as workbooks and summary sheets of the therapy aim and rationale to help consolidate learning may also be useful. It was also notable that VR was seen as something that could be helpful in many different ways on a ward, including for games or mindfulness exercises.

### Limitations

There were several limitations to the study. Most significantly, because of the COVID-19 pandemic, recruitment took place on only 3 acute psychiatric wards across 2 NHS mental health trusts, which may limit the generalizability of the findings. The enthusiasm for VR therapy may have been less on wards where, for example, there are more activities and therefore less boredom or on wards that already have psychological therapy available. It is also likely that participants in implementation studies may represent a more highly motivated group who are less representative of the whole population [16]. Multiple stakeholder involvement is important for implementation research [17,18], and there were too few staff participants (eg, consultants and managers) who are typically involved in strategic decision-making. It is also the case that this study did not consider all domains of the NASSS framework. For instance, it will also be valuable to consult individuals with detailed knowledge of the technology to consider supply, support, and future evolution. However, the results of this study indicate that VR therapy has significant potential to be implemented on psychiatric wards.

## Appendix

Multimedia Appendix 1 Extract from the coding and reflexive log.

## Abbreviations

LEAP: Lived Experience Advisory Panel
NASSS: nonadoption, abandonment, and challenges to the scale-up, spread, and sustainability
NHS: National Health Service
PIT: personal infrared transmitter
VR: virtual reality
