# Supplementary material for: Automated Virtual Reality Cognitive Therapy (gameChange) in Inpatient Psychiatric Wards: Qualitative Study of Staff and Patient Views Using an Implementation Framework
Source: JMIR Form Res. 2022 Apr 12;6(4):e34225. doi: 10.2196/34225 (PMC9044147; doi:10.2196/34225)
Supplement: Multimedia Appendix 1 [file formative_v6i4e34225_app1.pdf]

Extract from the coding and reflexive Log

A reflexive and coding log were kept throughout. This was firstly to consider how the authors' personal experiences and assumptions may be impacting the analysis, and secondly, to maintain a transparent audit trail. Below is an extract with illustrative examples of key considerations.

A preliminary thematic structure was developed by the lead author after the first two patient groups and the first two staff interviews had taken place. Discussion among the authorship team led to some reorganisation. A first example at the coding stage, was that there was initially a coding group called 'ward environment' with sub codes of such as medication, staff relationships, lack of therapy, and illness diversity. It was decided these should move to other relevant groups, for example, several were in reference to why there is a lack of therapy, and some were relevant to concerns about VR. These were therefore re-distributed accordingly. A second example, when considering high order themes, is that codes were initially structured into three overall themes: Pre-VR expectations and experiences, Thoughts after trying VR, and Future and implementation. Following reflection, it was noted these were more deductive than inductive, and while helpful for organising the coding log in NVivo, they did not capture the nuance of participants' views. A third example shows how discussion among the team led to a change in how a theme was described in the write up. Discussion was had around what the significance might be of the VR feeling much more real than participants expected. This led to a further review of the data, which highlighted that trying out the VR and experiencing its realism helped patients to understand how it could be helpful and reduced initial scepticism.

Attention was paid throughout to which ideas had been introduced to participants by the group facilitators and which had been spontaneously suggested by participants to ensure this was accurately reflected in the analysis. For example, the idea of 'peer professionals' was introduced to patients by the interviewers, where-as the idea of Nic being presented as someone with lived experience of severe mental health problems was suggested by one of the patients.

The thematic structure was then revisited upon completing data collection. Although many of the key themes remained the same, fleshed out with further examples and elaboration, a number of changes were also made. In particular, the extent of overlap between staff and patient themes became apparent, and it was therefore decided that the analysis could be written jointly.
